# Effects of Caffeine Supplementation on Power Performance in a Flywheel Device: A Randomised, Double-Blind Cross-Over Study

**DOI:** 10.3390/nu11020255

**Published:** 2019-01-24

**Authors:** Daniel Castillo, Raúl Domínguez, Alejandro Rodríguez-Fernández, Javier Raya-González

**Affiliations:** Facultad de Ciencias de la Salud, Universidad Isabel I, 09001 Burgos, Spain; daniel.castillo@ui1.es (D.C.); alejandro.rodriguez.fernandez@ui1.es (A.R.-F.); javier.raya@ui1.es (J.R.-G.)

**Keywords:** supplement, coffee, ergogenic aid, exercise, resistance exercise, resistance training, sport, squat, strength

## Abstract

Despite the demonstrated evidence of the importance of eccentric contractions in sports performance, no research has evaluated the ergogenic effects of caffeine on this type of contraction means during flywheel exercises. Therefore, the aims of the present study were to compare the power outcomes, using different inertial loads, between caffeine and placebo conditions. Twenty-four young, healthy, and active men (age: 22.5 ± 4.8 years) took part in the study. A crossed, randomised double-blind design was used to analyse the effects of caffeine on lower limb power outcomes during a flywheel half-squat exercise. Participants completed four sets of eight all-out repetitions with a fixed three-minutes rest interval, and each set was performed using different inertial loads (i.e., 0.025, 0.050, 0.075 and 0.100 kg·m^−2^). Both the mean power (MP) and peak power (PP) in concentric (CON) and eccentric (ECC) movement phases at each inertial load were recorded after participants were administered either a caffeine supplement (6 mg·kg^−1^) or placebo (sucrose). Participants receiving a caffeine supplementation demonstrated improvements versus the placebo in total MP (MP_total_), as well as MP in CON phase (MP_con_) and in ECC phase (MP_ecc_) at each inertial load (22.68 to 26.53%; *p* < 0.01, effect size (ES) = 0.89–1.40). In addition, greater improvements with caffeine ingestion were obtained with respect to the placebo condition (18.79 to 24.98%; *p* < 0.01, ES = 1.03–1.40) in total PP (PP_total_), as well as PP in CON phase (PP_con_) and in ECC phase (PP_ecc_) at each inertial load. Thus, the supplementation of 6 mg·kg^−1^ caffeine may be considered to maximise on-field physical performance in those sports characterised by high demands of resistance.

## 1. Introduction

The majority of sports, particularly team-based sports, are characterized by the need to apply the maximum force the athlete is able to produce over short time periods [1]. In this sense, it has previously been suggested that power outcomes are perhaps the most important characteristic impacting sport success [2], showing that a superior ability to generate maximal power typically results in enhanced athletic performance [3]. This fact appears to be evidenced by the high correlation between power outcomes and crucial short-term high intensity actions in sport performance, such as changes of direction, acceleration, deceleration, sprints or jumps [4]. These actions have been shown to be decisive in the achievement of success in team sports during competitions [5]. Along this line, increasing athletes’ power outcome levels could be an appropriate strategy to maximize on-field performance.

Nowadays, it is widely assumed that resistance training is the most extensively used method to improve athletes’ power production capacity to increase their physical condition [6]. Traditionally, free weights and weight stack machines have been used, but this equipment is only able to produce a high stimulus during the concentric (CON) phase, ignoring the eccentric (ECC) phase of the movement [7]. In this sense, isolated ECC actions during resistance programs are characterized by their production of higher dynamic resistance [8] with lower muscle activation and metabolic cost [9] and earlier increments in muscle mass [10], thus causing ECC actions to result in a potent stimulus for enhancements in neural and muscular adaptations [9], optimizing the effects of resistance training [11]. This has led to the use of this type of resistance strategy during athletes’ training periodization [10]. Attending to the aforementioned resistance training, the flywheel paradigm has been characterized by its ability to provide resistance through the inertial power generated by rotating flywheels during the acceleration and braking movements, in CON and ECC phases, respectively [12]. Due to the ECC load application, the flywheel paradigm has been shown to be an effective strategy to improve athletic performance in terms of increasing muscle mass [7], change of direction speed [13], jump height and sprint performance [14]. In this way, it has been shown that training by means of the flywheel paradigm has a positive effect on injury prevention [15].

Regarding athletic improvements in sport performance, a number of athletes previously consumed nutritional supplements [16]. However, only caffeine, creatine, β-alanine, sodium bicarbonate and beetroot juice have been proven to have an ergogenic or performance-enhancing effect based on scientific evidence [17]. Specifically, caffeine has proven to have a positive resistance performance effect [18,19,20], increasing the recruitment of motor units [21], improving the Na^+^–K^+^ pump response [22], and increasing the rate of calcium release from the sarcoplasmic reticulum [23], although some of these effects were only observed at toxic doses of caffeine [24]. A recent review [20] has suggested that caffeine ingestion may have a greater effect on contraction velocity rather than maximal force production. In this sense, previous studies have evaluated the effects of caffeine supplementation on power levels in resistance exercises [25,26,27,28,29], implying that caffeine supplementation (3 mg·kg^−1^) improves sports performance using loads corresponding to 25–75% of one maximum repetition (1RM) in the lower limbs [26,27,28]. However, positive effects in power have been observed using a dose of 6 mg·kg^−1^ in loads close to 1RM (90% 1RM) [28]. Given that only a few studies have examined the effects of caffeine ingestion on power outcomes in resistance exercise, future work is needed to elucidate this topic.

To address these research gaps, power outcomes during the CON and ECC phases were assessed in the present study in the flywheel half-squat exercise under two experimental conditions (i.e., placebo or caffeine). Specifically, the aim of the present study was to compare the power outcomes using different inertial loads (i.e., 0.025, 0.050, 0.075 and 0.100 kg·m^−2^) between caffeine and placebo conditions. We hypothesised, based on the presented scientific evidence [25,26,27,28], that caffeine supplementation is an effective strategy to achieve acute improvements in power outcomes during CON and ECC phases at each inertial load.

## 2. Materials and Methods

### 2.1. Participants

Twenty young, healthy and active men (age: 22.5 ± 4.8 years, height: 176.8 ± 5.7 cm, body mass: 73.1 ± 9.6 kg, and body mass index (BMI): 23.3 ± 2.5 kg·m^−2^;) took part in the study. All participants were team sport players and were actively training 3 ± 1 times per week, on average. In addition, participants had experience in systematic resistance training (at least one year), but none of them had previously trained using flywheel devices before the study. Participation was voluntary, and the following inclusion criteria were established in an informative session prior to the beginning of the study: (a) having completed, within the past 12 months, 1–2 sessions per week of strength training; (b) a half-squat 1RM greater than 1.5 times their body weight; (c) no intake of any nutritional supplements in the six months before the outset of the study; (d) no cardiovascular, respiratory, metabolic, neurological or orthopaedic disorders that could affect their half-squat performance; (e) not being a smoker; and (f) not being considered to be an elite athlete. In addition, a validated caffeine consumption questionnaire (i.e., caffeine consumption questionnaire) was administered to the participants, showing that all participants were light caffeine consumers (≤62 mg·day^−1^), since consumption is in the 20th percentile in a population with the same characteristics [30]. Furthermore, during this session, the researcher informed the participants of the test protocol, the study and its goals, and dietary guidelines to be followed during the investigation, including the intake of supplements. After their questions were resolved, participants signed written informed consent agreeing to participate in the study. This investigation met all the ethical principles of the declaration of Helsinki (2013), and the protocol was fully approved by the Ethics Committee of the Isabel I University (Burgos, Spain) prior to recruitment taking place.

### 2.2. Study Design

**Figure 1:**
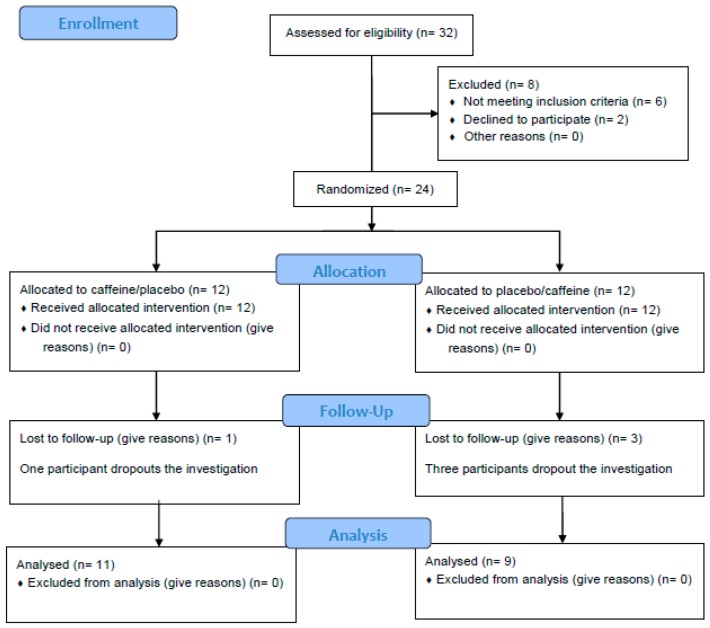
CONSORT diagram of the data collection process.

A crossed, randomised double-blind design was used to analyse the effects of caffeine on lower limb power outcomes during a flywheel half-squat exercise attending to different inertial loads (i.e., 0.025, 0.050, 0.075 and 0.100 kg·m^−2^). Participants reported to the sports performance laboratory to be tested four times over two consecutive weeks, with at least 72 h between consecutive sessions. A familiarisation period took place during the first two sessions, in order to avoid any learning effects [31], and the following two sessions corresponded to the experimental intervention. Upon arrival at the laboratory, participants were given a caffeine or a placebo supplement in a random, double-blind fashion. Forty-five minutes after the intake of the supplement, they started the experimental session. A lower limb power test was performed in a flywheel device to assess the power outcomes at each inertial load (i.e., 0.025, 0.050, 0.075 and 0.100 kg·m^−2^) on each testing day. Both the mean power (MP) and peak power (PP) values at each inertial load were recorded by a rotary encoder for further analysis. Participants were instructed to refrain from any type of physical exercise during the study and for 48 h before the first session. In each experimental session, 50% of the participants were subjected to different experimental conditions to avoid any effects of factor time or session on the results. For each participant, the sessions were carried out at the same time of day (± 0.5 h), in the mornings (from 10:00 a.m. to 1:00 p.m.), and under similar environmental conditions (21–23 °C). The data collection process (CONSORT diagram) is shown in Figure 1.

### 2.3. Nutritional Intervention and Diet Control

Participants were administered a caffeine supplement (6 mg·kg^−1^) or placebo (sucrose) dissolved in a 250 mL red bottle 35 min before starting the warm-up, to begin the power test alongside peak caffeine actions. As diet can affect energy metabolism during exercise, in the informative session participants were issued nutritional guidelines, to ensure that 48 h before each of the test sessions, they followed a similar diet consisting of 10% protein, 60% carbohydrates, and 30% lipids. Moreover, 24 h before each experimental session, participants’ intake of caffeine was restricted, and the participants were provided with a list of foods and drinks rich in caffeine (tea, coffee, mate, cola drinks, chocolate, energising drinks and chocolate drinks) that they should avoid. In addition, the last meal was eaten by participants 180 min before the test.

### 2.4. Lower Limb Power Test

**Figure 2:**
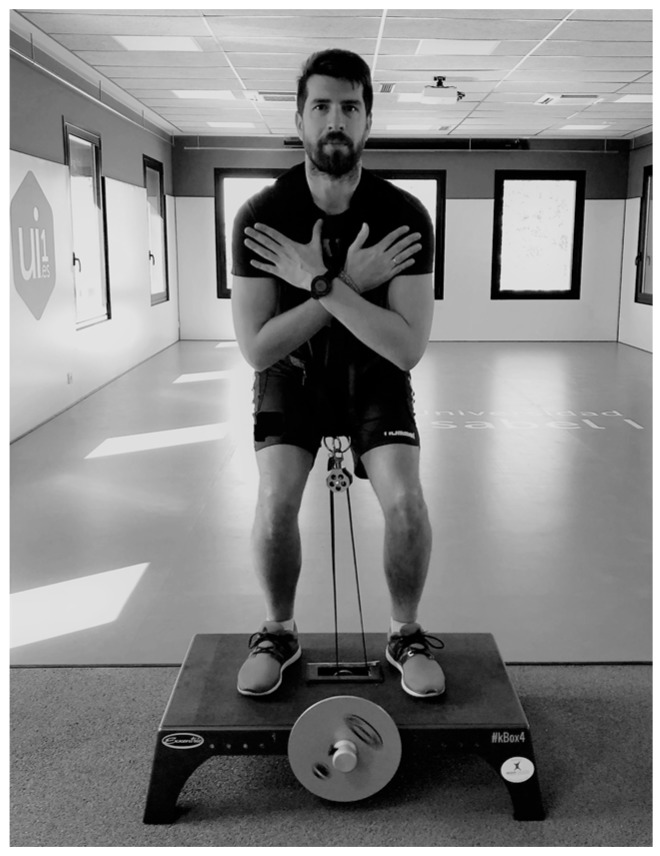
Description of the half-squat exercise using a non-gravity-dependent flywheel inertial device.

After a standardised 10-min warm-up, including cycling (5 min), joint mobility and dynamic stretching (3 min), as well as a submaximal set of eight repetitions of the half-squat flywheel exercise with an inertial load of 0.050 kg·m^−2^ (2 min), a lower limb power test was performed. The test consisted of the assessment of power in a half-squat exercise using a non-gravity-dependent flywheel inertial device (K-Box 4, Exxentric^®^, Stockholm, Sweden) (Figure 2), allowing participants to perform maximal CON and ECC actions [12]. This device is considered a valid and reliable tool to assess power outcome values in inexperienced participants [32]. Participants completed four sets of eight all-out repetitions with a fixed three-minutes rest interval, and each set was performed using different inertial loads: 0.025, 0.050, 0.075 and 0.100 kg·m^−2^. Both the first and second repetitions of each set were used to “increase velocity” and were excluded from data analysis [33]. To avoid possible fatigue effects, the participants were randomly divided into ascending order (starting with the lighter inertial load of 0.025 kg·m^−2^) and descending order (starting with the higher inertial load of 0.100 kg·m^−2^). Participants were required to perform the half-squat movement from the lower position (90°) to the full extension of the knees (180°), with no ankle extension allowed. The participants were instructed to perform the CON phase as fast as possible, while delaying the braking action to the last part of the ECC phase, with the aim of optimising ECC load production. Loud verbal encouragement was given to the participants during all testing sessions [33]. The MP and PP outcomes for the CON and ECC phases were sampled at 100 Hz [31] using an encoder compatible with the flywheel devices (SmartCoach™ Power Encoder SPE-35, SmartCoach Europe AB, Stockholm, Sweden) and an associated software (SmartCoach^®^ v.5.6.0.8, SmartCoach Europe AB, Stockholm, Sweden). In addition, the data analysis was performed using the mean of the six repetitions for each set.

### 2.5. Statistical Analysis

All results are provided as the mean (M) and standard deviation (SD). Normality was tested using the Shapiro–Wilk test, and statistical parametric techniques were applied. A *t*-test for paired samples was used to analyse the differences between the placebo condition and caffeine ingestion on MP and PP outcomes (i.e., total CON and ECC) for each inertia (i.e., 0.025, 0.050, 0.075 and 0.100 kg·m^−2^). The mean differences between the placebo condition and caffeine ingestion for each inertia were expressed as percentages:

mean difference (%) = ((Caffeine − Placebo)/Placebo) × 100.(1)

To allow a better interpretation of the results, the practical significance between the placebo condition and caffeine ingestion for each inertia was independently assessed by calculating Cohen’s *d* effect size [34]. Effect sizes (ES) of above 0.8, between 0.8 and 0.5, between 0.5 and 0.2, and lower than 0.2 were considered as large, moderate, small, and trivial, respectively. Significance was set at *p* < 0.05. All statistical tests were performed using the software package SPSS version 20.0 (SPSS Inc., Chicago, IL, USA).

## 3. Results

**Figure 3:**
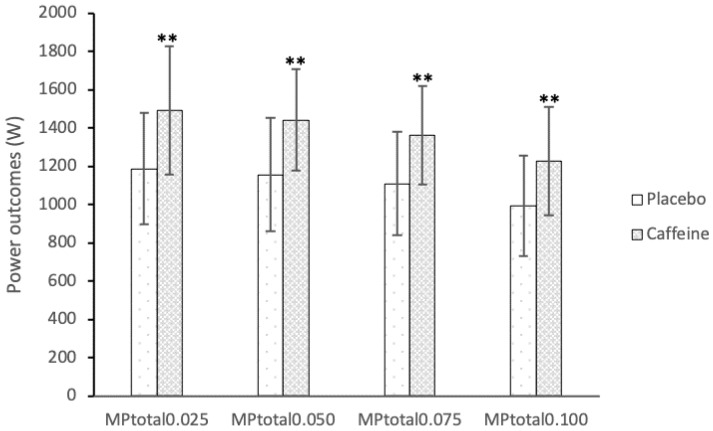
Effects of caffeine ingestion on total mean power (MP_total_) outcomes at different inertias when compared to the placebo condition. ** Significant differences (*p* < 0.01).

Caffeine supplementation demonstrated improvements in comparison to the placebo condition in total MP (MP_total_) 0.025 (1186.21 ± 291.16 vs. 1491.55 ± 334.42 W; ES = 1.05), MP_total_ 0.050 (1156.62 ± 295.39 vs. 1442.74 ± 266.51 W; ES = 0.97), MP_total_ 0.075 (1110.11 ± 267.96 vs. 1363.88 ± 256.57 W; ES = 0.95), and MP_total_ 0.100 (993.16 ± 262.19 vs. 1227.71 ± 284.03 W; ES = 0.89) (Figure 3).

In addition, Table 1 illustrates better MP_CON_ and MP_ECC_ outcomes for each inertia (i.e., 0.025, 0.050, 0.075 and 0.100 kg·m^−2^) with caffeine supplementation with respect to the placebo condition (*p* < 0.01, ES = 0.85–1.07).

**Figure 4:**
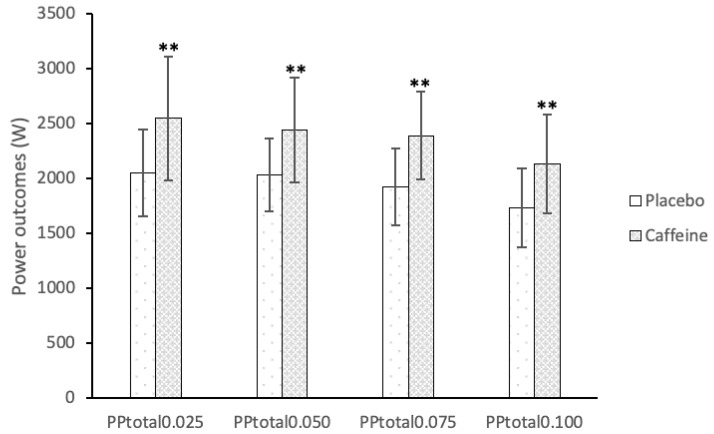
Effects of caffeine ingestion on total peak power (PP_total_) outcomes at different inertias when compared to the placebo condition. ** Significant differences (*p* < 0.01).

Caffeine supplementation also showed improvements in comparison to the placebo condition in total PP (PP_total_) 0.025 (2053.53 ± 397.24 vs. 2548.05 ± 562.09 W; ES = 1.24), PP_total_ 0.050 (2030.65 ± 334.43 vs. 2445.95 ± 477.69 W; ES = 1.24), PP_total_ 0.075 (1924.12 ± 349.76 vs. 2389.85 ± 402.60 W; ES = 1.33) and PP_total_ 0.100 (1735.74 ± 360.34 vs. 2130.85 ± 450.65 W; ES = 1.10) (Figure 4).

Likewise, Table 2 shows better PP_CON_ and PP_ECC_ outcomes for each inertia (i.e., 0.025, 0.050, 0.075 and 0.100 kg·m^−2^) with caffeine ingestion, with respect to the placebo condition (*p* < 0.01, ES = 1.03–1.40).

## 4. Discussion

The aim of the present study was to compare the power outcomes in a half-squat exercise using a flywheel device at different inertial loads (i.e., 0.025, 0.050, 0.075 and 0.100 kg·m^−2^) between caffeine and placebo conditions. The main findings of this study were as follows: (i) caffeine supplementation improved power outcomes significantly (*p* < 0.01) with respect to the placebo condition, with 25.7%, 17.9 %, 22.7% and 23.5% improvement for MP_total_ and 24.1%, 20.4 %, 24.2% and 22.7% for PP_total_ for 0.025, 0.050, 0.075 and 0.100 kg·m^−2^ inertial loads, respectively; (ii) caffeine supplementation significantly improved outcomes with respect to the placebo condition for both the MP_CON_ (24.5–26.5%; ES = 0.9–1.07) and MP_ECC_ (22.7–24.8%; ES = 0.85–0.98) at all inertial loads; (iii) caffeine supplementation significantly improved outcomes with respect to the placebo condition for both the PP_CON_ (22.12–24.16%; ES 1.14–1.40) and PP_ECC_ (18.79–24.98%; ES = 1.03–1.17) for all the inertial loads used.

Research has shown that caffeine supplementation of 3 mg·kg^−1^ effectively increases average power levels by 4.6–6.9% at 75% 1RM [27], and 6.9% during an incremental test (10%) until reaching 1RM [25]. In addition, improvements of 5.4–8.5% [27] and 6.3–8.9% [28] at 75–90% 1RM in half-squat exercises have been observed by the supplementation of 6 mg·kg^−1^ caffeine. The improvements obtained in our investigation were higher than the aforementioned studies (~25% vs. 4.6–8.9%) using traditional half-squat exercises. These differences could be explained by the nature of flywheel exercises, in which an accentuated ECC overload [35] and higher muscle activation during CON actions [36] are induced. Therefore, it is suggested that caffeine supplementation, between 3–6 mg·kg^−1^, effectively elicits higher power outcomes in short-terms situations, and so it is advised that it be used to maximise on-field physical performance in sports characterised by high demands of resistance.

The improvements after caffeine supplementation in MP_total_, MP_CON_, MP_ECC_, PP_total_, PP_CON_ and PP_ECC_ can be explained through several mechanisms, among which the performance of caffeine on the A_1_, A_2A_ and A_2B_ adenosine receptors stands out [37]. During physical exercise, adenosine inhibits the activity of efferent nerves whilst stimulating afferent nerves [38]. Therefore, caffeine stimulates the synthesis of neurotransmitters with a cerebral excitatory character that causes an increase in arousal and mood by means of an improvement in vigour [39] and tension [40]. According to the governor theory proposed by Noakes et al. [41], stimuli can produce electrical signals in the brain that lead to fatigue during exercise, which could have a positive effect on performance. In addition, through the mediation of adenosine antagonism, caffeine supplementation may have increased neuromuscular recruitment [42]. Because this increase is accompanied by an improvement in muscle strength, the time needed to reach the maximum power levels is reduced [21], which would have positively influenced the higher levels of power reported in our results. Given that producing greater power outcomes in short time periods is related to an improvement in the team sports performance [1], coaches could consider administering caffeine supplementation prior to the official competitions.

Previous research has reported that during resistance training sessions, caffeine supplementation increased the maximum number of repetitions realised with submaximal loads [43]. Also, in our investigation, caffeine supplementation elicited higher power outcomes in comparison to the placebo condition. In our study, participants performed four sets of eight all-out repetitions using a device that produced a high load in both CON and ECC movement phases [31], which gives a high-intensity character to this type of resistance training [12]. In this way, it is possible that the effect of catecholamines would have favoured the increase on performance after caffeine supplementation because catecholamines increase the activity of the central and peripheral nervous system. In addition, our results demonstrated an increase in ECC power after caffeine supplementation. These findings could be because fast fibres present a greater liberation of calcium and caffeine, which has been shown to increase the release of calcium [23]. In a practical approach, those athletes who would be able to exhibit higher values of ECC power could have a reduced injury risk during short-term and high-intensity actions, because their lower-limb muscles would be able to absorb more energy during the ECC phase. This is explained by fact that the area under the length–tension deformation curve is larger, what could help to reduce the lower-limb muscle injury risk [44]. Thus, coaches could consider the supplementation of caffeine as an additional strategy to enhance higher ECC power outcomes.

This study is not exempt of limitations, the main one being that our results cannot be generalized to upper body musculature, given that the effects of caffeine may not be uniform in both upper and lower body. Neither can our findings can be generalized to female athletes, because only men participated in the investigation. In addition, in our study participants did not ask about the supplementation administered in each session, so they were not allocated depending on their response [45]. Finally, the results for plasma caffeine, epinephrine, and norepinephrine concentration have not been determined. On the other hand, the main strength of our investigation is that the flywheel device used allows us to measure the power generated in a specific speed context, not only in the CON phase but also in the ECC phase. Besides, the effects of caffeine supplementation have been assessed at different inertias, which involves a further knowledge about the influence of caffeine supplementation on the half-squat performances.

## 5. Conclusions

Caffeine supplementation is an effective strategy to increase MP_total_, MP_CON_, MP_ECC_, PP_total_, PP_CON_ and PP_ECC_ outcomes during a half-squat exercise by means of a flywheel device. In this sense, the application of 6 mg·kg^−1^ of caffeine may be considered to maximise on-field physical performance in those sports characterised by high demands of resistance.

## Figures and Tables

**Table 1:** Effects of caffeine ingestion on concentric (CON) and eccentric (ECC) mean power (MP) outcomes (W) at different inertias compared to the placebo condition.

Variable	Placebo (M ± SD)	Caffeine (M ± SD)	Mean Differences (%)	ES	*p*
MP_CON_0.025	629.57 ± 155.79	796.63 ± 170.76	26.53	1.07	0.00
MP_ECC_0.025	556.64 ± 141.52	694.92 ± 172.72	24.84	0.98	0.00
MP_CON_0.050	586.31 ± 139.49	733.01 ± 146.09	25.02	1.05	0.00
MP_ECC_0.050	570.30 ± 162.39	709.72 ± 136.92	24.45	0.86	0.00
MP_CON_0.075	542.05 ± 120.64	665.00 ± 131.92	22.68	1.02	0.00
MP_ECC_0.075	568.06 ± 153.69	698.87 ± 134.11	23.03	0.85	0.00
MP_CON_0.100	471.15 ± 127.45	586.91 ± 132.33	24.57	0.91	0.00
MP_ECC_0.100	522.01 ± 138.75	640.80 ± 157.15	22.76	0.86	0.00

M: mean; SD: standard deviation; ES: effect size.

**Table 2:** Effects of caffeine ingestion on concentric (CON) and eccentric (EEC) peak power (PP) outcomes (W) at different inertias compared to the placebo condition.

Variable	Placebo (M ± SD)	Caffeine (M ± SD)	Mean Differences (%)	ES	*p*
PP_CON_0.025	1067.67 ± 216.94	1325.57 ± 269.82	24.16	1.19	0.00
PP_ECC_0.025	985.86 ± 185.63	1222.48 ± 310.17	24.00	1.17	0.00
PP_CON_0.050	1014.37 ± 178.03	1238.73 ± 245.63	22.12	1.26	0.00
PP_ECC_0.050	1016.29 ± 174.29	1207.22 ± 262.78	18.79	1.10	0.00
PP_CON_0.075	927.37 ± 154.70	1144.14 ± 206.49	23.37	1.40	0.00
PP_ECC_0.075	996.74 ± 213.39	1245.71 ± 210.09	24.98	1.17	0.00
PP_CON_0.100	817.55 ± 170.71	1012.69 ± 210.84	23.87	1.14	0.00
PP_ECC_0.100	918.18 ± 194.27	1118.17 ± 245.91	21.78	1.03	0.00

M: mean; SD: standard deviation; ES: effect size.
